# Radix Astragali and Its Representative Extracts for Diabetic Nephropathy: Efficacy and Molecular Mechanism

**DOI:** 10.1155/2024/5216113

**Published:** 2024-09-14

**Authors:** Hui-zhong Xue, Yu Chen, Shi-dong Wang, Yi-meng Yang, Lu-qi Cai, Jin-xi Zhao, Wei-jun Huang, Yong-hua Xiao

**Affiliations:** ^1^ The First Clinical Medical School Beijing University of Chinese Medicine, Beijing, China 100700; ^2^ Key Laboratory of Chinese Internal Medicine of Ministry of Education and Beijing Dongzhimen Hospital Beijing University of Chinese Medicine, Beijing, China 100700; ^3^ Section II of Endocrinology & Nephropathy Department Dongzhimen Hospital Beijing University of Chinese Medicine, Beijing, China 100700

**Keywords:** diabetic nephropathy, efficacy, pharmacological mechanism, Radix Astragali, traditional Chinese medicine

## Abstract

Diabetic nephropathy (DN) is a common microvascular complication of diabetes mellitus (DM). Radix Astragali (RA), a frequently used Chinese herbal medicine in the Leguminosae family, *Astragalus* genus, with its extracts, has been proven to be effective in DN treatment both in clinical practice and experimental studies. RA and its extracts can reduce proteinuria and improve renal function. They can improve histopathology changes including thickening of the glomerular basement membrane, mesangial cell proliferation, and injury of endothelial cells, podocytes, and renal tubule cells. The mechanisms mainly benefited from antioxidative stress which involves Nrf2/ARE signaling and the PPAR*γ*-Klotho-FoxO1 axis; antiendoplasmic reticulum stress which involves PERK-ATF4-CHOP, PERK/eIF2*α*, and IRE1/XBP1 pathways; regulating autophagy which involves SIRT1/NF-*κ*B signaling and AMPK signaling; anti-inflammation which involves IL33/ST2 and NF-*κ*B signaling; and antifibrosis which involves TGF-*β*1/Smads, MAPK (ERK), p38/MAPK, JNK/MAPK, Wnt/*β*-catenin, and PI3K/AKT/mTOR signaling pathways. This review focuses on the clinical efficacy and the pharmacological mechanism of RA and its representative extracts on DN, and we further document the traditional uses of RA and probe into the TCM theoretical basis for its application in DN.

## 1. Introduction

Diabetic nephropathy (DN), also known as diabetic glomerulosclerosis, is a series of renal pathological changes resulting from diabetes mellitus (DM), including glomerular basement membrane (GBM) thickening, mesangial expansion, extracellular matrix (ECM) deposition (mainly mesangial), and tubular atrophy, leading to renal interstitial fibrosis and glomerulosclerosis [1–3]. As one of the most prevalent diabetic microvascular complications, DN has become the leading cause of end-stage kidney disease (ESRD) [3]. It is clear that mechanisms like oxidative stress, endoplasmic reticulum (ER) stress, inflammation, and autophagy impairment are involved in the pathological changes aforementioned [1]. However, the treatment of DN is still mainly limited to the control of blood glucose and blood pressure so far [4].

For DN treatment, Chinese herbal medicine (CHM) shows the modulative function of metabolism, inflammation, oxidative stress, ER stress, and fibrosis and has been proven useful in clinical practice for glycosylated hemoglobin (HbA1c), fasting blood glucose (FBG), urinary albumin excretion rate (UAER), serum creatinine (Scr), 24-h urinary protein (24 h UP), and estimated glomerular filtration rate (eGFR) [5]. Obviously, CHM is a promising therapeutic tool in DN treatment.

Radix Astragali (RA) is one of the most widely used herbs for the traditional Chinese medicine (TCM) treatment of DN, which is the dry root of *Astragalus mongholicus* Bunge or *Astragalus membranaceus* (Fisch.) Bunge [6]. It was first recorded in the book *Prescriptions of Fifty-Two Diseases*, written in the Warring States period (475-221 BC). Its main therapeutic effect is tonifying qi. The main pharmacological components of RA include polysaccharides, saponins, and flavonoids [7]. Among these components, astragalosides take the highest proportion of triterpene saponin content in RA [7]. As the marking compound of RA, astragaloside IV (AS-IV) has been documented with the functions of neuroprotection, liver protection, hematopoietic system protection, antivirus, antibacteria, immunity enhancement, antitumor, and antidiabetes and its complications [8, 9]. In addition, as the highest content component of RA, astragalus polysaccharide (APS) has also been widely studied, including the efficacy of immune regulation, antiaging, antitumor, reducing blood glucose and blood lipid, antifibrosis, antibacterial, antivirus, and radiation protection [10]. Flavonoid compounds in RA are mostly calycosin-7-O-*β*-D-glucoside, calycosin, ononin, and formononetin. Calycosin-7-O-*β*-D-glucoside and calycosin are the representative constituents of RA and have been proven with antitumor, neuroprotective, antidiabetic, antiosteoporosis, liver protection, and cardiovascular system protection effects [11, 12]. With the extensive efficacy of these components above, recent research has provided insight into the value of RA in the treatment of tumors, coronary heart disease, diabetes, nephritis, and so on [6, 9, 10, 12].

## 2. Therapeutic Effects of RA on DN

### 2.1. Clinical Efficacies

For DN, the therapeutic effect of RA has been proved by many clinical trials. A meta-analysis of 21 RCTs and 4 CCTs with 1804 subjects assessed the efficacy of intravenous drip of RA injection in treating Stages III–IV (Mogensen) DN. Results showed that RA injection contributed to a better effect in decreasing blood urea nitrogen (BUN), Scr, creatinine clearance rate (Ccr), and urinal protein, and increasing serum albumin (ALB) level compared with the control group (ACEi/ARB) [13]. The updated meta-analysis of 66 RCTs with 4785 patients (no restriction on the stage of renal function) including all forms of RA preparation (including tablet, granule, decoction, extract liquid, and injections) showed that adjunctive use of RA preparations on conventional therapies of ACEi/ARB could reduce albuminuria, proteinuria, and Scr in DN patients [14]. A retrospective cohort study among 6648 predialysis DN patients found RA users with lower all-cause mortality compared with other CHM users after a 5-year follow-up [15]. Here, we summarize the study design, main conclusions, and safety outcomes of the clinical research on RA in treating DN in the past 10 years (see Table 1). The preparation mostly used is RA injection (from 20 to 50 mL qd). RA tablets (2.2 g, bid), granules (from 4 to 15 g, qd or bid), and APS injection (250 mg, qd) are also common preparations and dosages. These indicate that the optimal dose for RA injections and oral preparations has not been fully validated, and whether the injection or the oral preparations can give play to higher effects still needs investigation. The frequent adverse effects (AEs) in these studies include gastrointestinal reactions, liver function damage, hyperkalemia, cough, dizziness, and headache. Parts of these AEs are common in ACEI/ARBs treatment, and no statistical difference was reported in the incidences of AEs between the treatment group and the control group. Therefore, it is not yet clear about the AEs of RA preparations. Besides, 31 out of these 50 researches did not report safety outcomes, suggesting insufficient attention to AEs of TCM. Anyhow, RA preparations exert a therapeutic effect on DN by reducing urinary protein and decreasing BUN, Scr, and Ccr. These finally result in a reduction of endpoint events, indicating the supplementary treatment value of RA for DN.

### 2.2. Histological Effects

**Figure 1:**
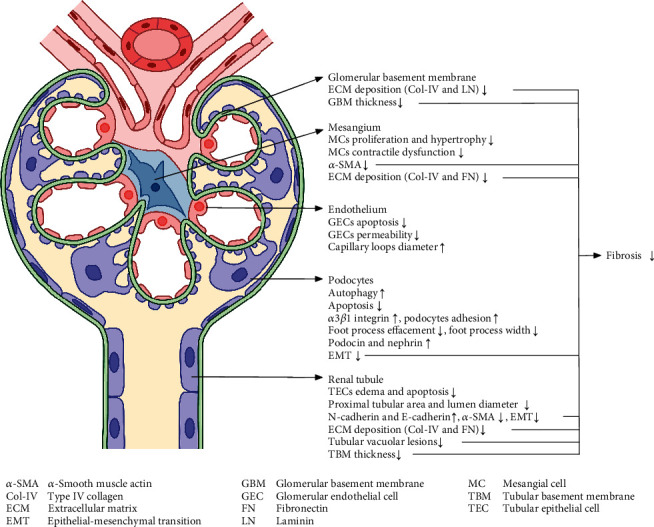
Main pathological effects of RA against DN. The renal protective effect of RA targets multiple parts of the nephron, including the glomerular basement membrane, mesangium (both mesangial cells and mesangial matrix), endothelium, podocytes, and tubule. Note: Symbols “↑” and “↓” represent increase and decrease, respectively.

When injuries occur to renal cells, the responses vary from hypertrophy, proliferation, activation, and transformation to necrosis and apoptosis. These cells can secrete a variety of cytokines and inflammatory mediators and produce varying amounts of ECM, resulting in glomerulosclerosis and tubulointerstitial fibrosis; this is how renal function is damaged [66]. If we can balance cell proliferation and apoptosis, control cell activation, and phenotypic transformation, it will greatly suppress the histopathology changes of renal tissue and increase the possibility of curing and delaying nephropathy. Therefore, it is of great significance to study the pathological effects in the treatment of disease and injury. Based on the current studies, RA has shown protective effects on specific renal intrinsic cells and emerged as a promising treatment through the modulation of multiple pathogeneses in DN progress, especially in renal fibrosis (Figure 1). Detailed effects include reducing ECM deposition; alleviating GBM and TBM thickening; ameliorating mesangial cell (MC) proliferation, hypertrophy, and contractile dysfunction; alleviating mesangial hyperplasia; inhibiting glomerular endothelial cell (GEC) apoptosis; restoring increased GEC permeability and capillary loop diameter and decreased podocyte autophagy, podocin, and nephrin expression; suppressing podocyte apoptosis, detachment, and foot process effacement; decreasing foot process width; reversing promoted epithelial-mesenchymal transition (EMT) in podocytes and renal tubular epithelial cells (TECs) (RTEC); inhibiting TECs' edema and apoptosis; and so on.

## 3. Mechanisms of RA and Its Representative Extracts on DN

RA and its representative extracts exhibit the renal protective effect in DN through multiple mechanisms independent of antihyperglycemic.

### 3.1. Inhibition of Oxidative Stress

When a free radical, independent chemical species with one or more unpaired electrons, attacks a nonradical molecule, a secondary radical molecule comes into being. The chain reaction of primary and secondary radicals leads to oxidative damage of tissues, while antioxidants can block the initial production of free radicals and the secondary production of toxic metabolites. When excessive oxidant compounds and insufficient antioxidants lead to an imbalanced redox state, this is what we call oxidative stress [67]. Hyperglycemia promotes the generation of advanced glycation end products (AGEs) in the cells, which trigger mitochondria to release excess reactive oxygen species (ROS), resulting in renal inflammation, renal cell injury, and fibrosis [5, 68].

The astragalosides in RA, mostly AS-IV, exhibit significant antioxidant effects. In diabetic db/db mice, AS-IV ameliorated podocyte injury by enhancing klotho expression and inhibiting oxidative stress through the PPAR*γ*-Klotho-FoxO1 axis [69]. In STZ-induced hyperinsulinemia rats, AS-I could decrease the level of AGEs [70] and suppress the activation of nicotinamide adenine dinucleotide phosphate (NADPH) oxidase 4 (Nox4) expression and the phosphorylation of ERK1/2. These actions further reduce malondialdehyde (MDA) and increase activities of antioxidant enzymes such as glutathione (GSH) peroxidase (GSH-Px), superoxide dismutase (SOD) [71, 72], and catalase (CAT) [73] and increase total antioxidative capability (T-AOC) [70], thus inhibiting oxidative stress.

In vitro results were consistent with those from animal studies. AS-IV reduced the content of ROS [73], restored Klotho expression, enhanced forkhead box transcription factor O1 (FoxO1)-dependent antioxidant activity, and upregulated Nrf2-ARE/TFAM signaling to resist oxidative stress damage, protecting HG-exposed podocytes from apoptosis [69, 72]. AS-IV also inhibited MC proliferation and glomerular contractile dysfunction caused by HG culture. These effects were achieved through the NADPH oxidase/ROS/Akt/NF-*κ*B pathway [74, 75]. The inhibitory effect of AS-IV on oxidative stress can also protect renal tubular cells. Qi et al. [76] proved that AS-IV could attenuate glycosylated albumin (GA)-induced amplification of ROS, inhibit NADPH oxidase activity, and elevate the level of SOD units in the NRK-52E cell line. In HG-exposed HK-2 cells, AS-IV could regulate the Wnt/*β*-catenin pathway and Nrf2/ARE signaling pathway, an endogenous antioxidant stress pathway; further enhance the activities of SOD, GSH-Px, and CAT; and decrease the generation of MDA and ROS [77, 78]. Another study on HG-induced HK-2 cells also found that AS-IV could alleviate palmitic acid (PA)-induced cell apoptosis by decreasing ROS production and increasing P-Nrf2 levels [79]. These antioxidant processes lead to the prevention of cell apoptosis, which might be associated with the reduction of apoptotic proteins like Bax, cleaved-caspase-3, cleaved-caspase-9, and Bcl-2 [73, 77, 79].

The antioxidant effect of calycosin and calycosin-7-O-*β*-D-glucoside, which are also representative constituents of RA, has also been proved. In human GECs under HG stimulation, calycosin and calycosin-7-O-*β*-D-glucoside could attenuate AGEs-mediated cell apoptosis [80]. In NRK-52E rat renal TECs, calycosin recovered HG-induced cell viability decrease and membrane damage, reduced ROS generation and lipid peroxidation, increased the expression of (sirtuin-3) SIRT-3/SOD2 at both mRNA and protein levels, and inhibited the activity of caspase-3 [81]. In the high-fat diet(HFD)/STZ-induced T2DM rats model, calycosin treatment decreased ROS, MDA, and thioredoxin-interacting protein (TXNIP) levels, while elevated Nrf2 and TAC levels recovered mitochondrial viability and mitochondrial respiration dysfunction, indicating its potential for alleviating oxidative stress [82–84].

### 3.2. Inhibition of ER Stress

The main physiological function of the ER is to synthesize proteins. Pathophysiological stress conditions may interfere with normal protein folding and cause accumulation and aggregation of misfolded and unfolded proteins (UPR), inducing cellular toxicity. ER stress occurs when excess UPR accumulates beyond the load that the cell can deal with [85]. HG condition leads to altered protein glycosylation, which can interfere with protein folding, causing the accumulation of misfolded and UPR in the ER and contributing to ER stress, which could be cytotoxic after prolonged processes [85, 86].

In HFD and STZ-induced DN rats, intragastric AS-IV could alleviate RTEC apoptosis by downregulating the expression of ER stress-related proteins like p-PERK, ATF4, and CHOP and restoring the balance of Bax and Bcl-2 expression [87]. In STZ-induced DN rats, AS-IV could also alleviate ER stress-induced podocyte apoptosis by suppressing the PERK-ATF4-CHOP pathway [88]; inhibiting the expression of ORP150 and GRP78; and downregulating the phosphorylation of PERK, eIF2*α*, and JNK [89]. In db/db mice and podocytes under HG or PA stimulation, AS-IV ameliorated podocyte apoptosis by attenuating ER stress by restoration of SERCA2 expression and activation [90, 91]. Overall, AS-IV exerts its anti-ER stress effect by blocking the PERK-ATF4-CHOP, PERK/eIF2*α*, and IRE1/JNK pathways; upregulating SERCA2 expression; and downregulating ORP150 and GRP78 expression.

### 3.3. Control of Regulated Cell Death (RCD)

Physiologically, RCD clears unwanted cells, but in some pathological situations, disproportionate responses can contribute to the detrimental loss of kidney cells [92]. Based on molecular and essential aspects of the cell death process, the RCD process can be categorized into different modalities such as intrinsic apoptosis, extrinsic apoptosis, MPT-driven necrosis, necroptosis, ferroptosis, pyroptosis, parthanatos, entotic cell death, NETotic cell death, lysosome-dependent cell death, autophagy-dependent cell death, and immunogenic cell death.

Pyroptosis is triggered by perturbations of extracellular or intracellular homeostasis related to innate immunity manifesting with a peculiar form of chromatin condensation as well as cellular swelling culminating with plasma membrane permeabilization [93]. Pyroptosis generally relies on the activation of one or more pyroptotic caspases (caspase-1, caspase-4, caspase-5, and caspase-11) and is always associated with the activation and secretion of the two prominent proinflammatory cytokines, interleukin-1*β* (IL-1*β*) and IL-18 [94]. In the db/db mouse model, AS-IV inhibited the expression levels of NOD-like receptor thermal protein domain associated protein 3 (NLRP3) inflammasome, apoptosis-associated speck-like protein (ASC), caspase-1, and IL-1*β* in the renal cortex and reduced the serum levels of TNF-*α* and monocyte chemoattractant protein-1 (MCP-1), thus protecting podocytes from pyroptosis accompanied by the upregulation of the podocyte markers podocin and synaptopodin [95, 96]. In STZ-induced rats, oral treatment with calycosin decreased Scr, BUN, and proteinuria levels; escalated urine Ccr; and alleviated interstitial collagen deposition and GBM thickening. These alternations were accompanied by decreased levels of serum LDH and IL-1*β*, renal NF-*κ*Bp65, pyroptotic-related proteins (TXNIP and NLRP3), and MDA and elevated IL-10, TAC, and Nrf2 expression, indicating that the renal-protective effect of calycosin might be associated with the modulation of oxidative stress NLRP3 and TXNIP-mediated pyroptotic signaling [84].

Ferroptosis is a form of RCD characterized by the iron-dependent accumulation of lipid hydroperoxides. It could be induced by the accumulation of glutamate, iron, or polyunsaturated fatty acid phospholipids, or by depletion of endogenous inhibitors of ferroptosis, such as reduced GSH, NADPH, Phospholipid Peroxidase Glutathione Peroxidase 4 (GPX4), or vitamin E [97]. Calycosin has been proven to be able to regulate ferroptosis in diabetic kidney disease through in vivo and in vitro studies. In db/db mice, it decreased BUN and Cr, alleviated tubular dilatation and collagen deposition, and reversed the loss of the proximal tubule brush border. In HG-treated HK-2 cells, it increased cell viability. These were achieved by the upregulation of GPX4, inhibition of lipid ROS production, and nuclear receptor coactivator 4 (NCOA4) expression, which are all correlated with ferroptosis [98].

Autophagy, a process of degrading damaged proteins and macromolecules, is a cellular protective mechanism. The autophagy levels of renal cells (including podocytes, proximal renal tubules, MCs, and endothelial cells) are downregulated in hyperglycemia conditions, which can promote the development of DN [99]. AS-IV can restore the autophagy of podocytes by inhibiting AMPK*α* activation, as proved by the upregulation of autophagy-related proteins LC3A/B, Beclin-1, and autophagy-related protein 12 (Atg12) and downregulation of autophagy-related protein p62 (p62) [90]. In MCs, AS-IV can also regulate Beclin-1 and LC3 II through SIRT1/NF-*κ*B signaling, subsequently suppressing MC proliferation [100].

### 3.4. Anti-Inflammatory Effect

Diabetes can be regarded as chronic low-grade inflammation or a preinflammatory state. Inflammatory cytokines, such as TNF-*α*, IL-1*β*, and IL-6, activate a variety of signal pathways, resulting in insulin resistance and decreased insulin secretion, and promote the development of diabetes [101].

Studies suggested that AS-IV could restore IL-1*β*, IL-4, IL-6, TNF-*α*, MCP-1, and ICAM-1 overproduction in STZ-induced rats, indicating its effect on ameliorating renal injury by inhibiting NF-*κ*B-mediated inflammatory gene expression [71, 78, 102]. APS and calycosin could also ameliorate inflammation in DN kidneys by suppressing the NF-*κ*B-dependent signaling pathway, confirmed by the downregulation of Toll-like receptor 4 (TLR4) and the phosphorylation of I*κ*B*α* and NF-*κ*B p65 and the expression of TNF-*α*, IL-1*β*, and MCP-1 in diabetic rats/mice, AGEs-treated mouse TECs, and HG-treated mouse podocytes [103–105]. Moreover, in the HFD/STZ-induced T2DM rat model, calycosin has shown the ability to modulate IL33/ST2 signaling and decrease levels of inflammatory cytokines such as NF-*κ*B p65, TNF-*α*, and IL-1*β* [82].

### 3.5. Inhibition of Renal Fibrosis

Renal fibrosis is characterized by tubule atrophy, interstitial chronic inflammation and fibrogenesis, glomerulosclerosis, and vascular rarefaction [106]. Abnormal activation of several cell growth factors such as transforming growth factor beta (TGF-*β*), connective tissue growth factor (CTGF), angiotensin II (Ang II), hepatocyte growth factor (HGF), and their receptors are the mediators of renal fibrosis [107, 108].

In the human glomerulus, known cell types include GECs, podocytes, MCs, and parietal epithelial cells. Podocyte injury results in their detachment and loss, which is one of the major determinants of glomerular injury and glomerulosclerosis. The manner in which endothelial cells contribute to glomerulosclerosis is to regenerate and produce GBM, causing the thickening of GBM. Similarly, the pathological changes of MCs and parietal epithelial cells during fibrosis progress are to regenerate, proliferate, and produce ECM, which also potentially leads to glomerulosclerosis [106, 109].

As for the renal tubule, in the DN process, infiltrating immune cells, glucosuria, and albuminuria can trigger the secretion of proinflammatory and profibrotic mediators in proximal TECs, resulting in interstitial inflammation and fibrosis [110]. EMT is one of the pathognomonic pathological processes of interstitial fibrosis, which manifests as the loss of the original phenotype of TECs, the destruction of the renal tubular basement membrane, the shift of TECs from the renal tubules to the interstitium through the damaged basement membrane, and the transformation of TECs into myofibroblasts expressing *α*-smooth muscle actin (*α*-SMA) [108]. Another typical pathological change of interstitial fibrosis is ECM accumulation, which represents the excess accumulation of matrix proteins including Types *Ι*, III, V, VI, VII, and XV collagen, as well as the adhesive glycoprotein fibronectin (FN), which results in the expansion of the space between the tubular basement membrane and peritubular capillaries [111].

Vitro experiments on HK-2 and NRK-52E cell lines have confirmed that AS-IV can reverse the HG or GA-induced increase in the expression of *α*-SMA and decrease the expression of E-cadherin to attenuate EMT [76, 112]. In MCs under HG condition, both AS-IV and astragalosides could inhibit increased cell proliferation; downregulate the expression of CTGF, TGF-*β*1, and fibrosis-related mRNAs including miR-192 and miR-21; alleviate the increased levels of *β*-catenin and Smad3; decrease the level of Smad7 regulated by miR-21 overproduction; and reduce major ECM proteins including Type IV collagen (Col-IV) and FN [113–115]. In HG-exposed TECs, AS-IV could also decrease the production of *β*-catenin, TGF-*β*1, *α*-SMA, Col-IV, and FN and increase E-cadherin expression, antagonizing EMT and renal fibrosis [78, 116, 117]. In mouse renal fibroblasts cultured with TGF-*β*1, AS-IV treatment suppressed the cell viability and the overexpression of *α*-SMA, Col-IV, and FN, further inhibiting fibroblast differentiation and ECM formation [118]. These effects were reached probably through Wnt/*β*-catenin, TGF-*β*1/Smads, mTORC1/p70S6K, MAPK, NF-*κ*B, and RAF/MEK/ERK pathways [78, 112–118].

AS-IV and AS-I also exhibited antifibrosis effects on STZ-induced DN rats, as proved by the amelioration of glomerular mesangial hyperplasia and GBM thickening [70, 114]. Mechanisms include the inhibition of the expression of TGF-*β*1 and its downstream mediator Smad3, increase of the expression of Smad7 (which can negatively regulate Smad3), and downregulation of miR-192, *α*-SMA, and Type 1 collagen (Col-1) expression via the TGF-*β*1/Smad/miR-192 signaling pathway [114]. Studies of AS-IV and astragaloside on diabetic KKAy mice decreased *α*-SMA, Col-IV, and FN expression and showed suppression effect of glomerulosclerosis and renal interstitial fibrosis accompanied by the downregulation of miR-21, TGF-*β*1, Smad2/3, and *β*-catenin [115, 119]. These results also proved that the TGF-*β*/Smads and Wnt/*β*-catenin pathways were involved in the antifibrosis effect of RA. In diabetic mouse models, AS-IV also exhibited its effect of ameliorating mesangial expansion, reducing ECM deposition through inhibition of CX3CL1-RAF/MEK/ERK, MEK1/2ERK1/2-RSK2, Akt/mTOR, NF-*κ*B, and Erk1/2 signaling pathways [112, 120, 121].

Additionally, in vitro and in vivo studies demonstrated that calycosin treatment could inhibit renal ECM deposition and modulate fibrotic processes [80, 82].

### 3.6. Other Mechanisms Against DN

Hyperglycemia-induced GEC injury manifests as cell dysfunction, even apoptosis. Endothelial dysfunction in diabetes is associated with impaired abilities of nitric oxide (NO) production and eNOS activation [122]. AS-IV has been proven to be able to promote the synthesis of NO, enhancing eNOS phosphorylation and activity, thereby reversing HG-treated human renal GEC permeability and apoptosis [123].

The pathological changes of podocytes in DN include hypertrophy, dedifferentiation (EMT), impaired autophagy, podocyte detachment, effacement, and apoptosis [68]. *α*3*β*1 integrin acts as an anchor to connect the podocyte with the GBM. AS-IV can increase the expression of *α*3*β*1 integrin and decrease the expression of integrin-linked kinase (ILK), subsequently ameliorate podocyte adhesion dysfunction, inhibit podocyte detachment [124], and increase podocyte density [125]. TRPC6, one of the Ca^2+^ ion channels expressed in podocytes, is involved in NFAT-dependent cell apoptosis. AS-IV can prevent HG-exposed podocyte apoptosis via downregulation of TRPC6, NFAT2, and Bax expression [126]. Apoptosis of podocytes is also associated with TRAF5-mediated NF-*κ*B activation [127]. AS-IV increases the expression of miR-378 and elevates the TUG1 level, both of which can attenuate the TRAF5 level, thus suppressing podocyte apoptosis in DN rats and HG-treated MPC5 cells [128, 129].

Adiponectin is an adipose-derived hormone. It has proven to be beneficial in DN by means of a comprehensive effect of antioxidation, anti-inflammatory, modulating RCD, anti-ER stress, antifibrosis, and improving endothelial dysfunction. However, during the progress of glucose and lipid metabolism disorders, the secretion of adiponectin is downregulated [130–132]. In a db/db mouse model, HG-treated human GECs, and murine podocytes, AdipoRon, a synthetic adiponectin receptor agonist, showed the ability to activate intrarenal AdipoR1 and AdipoR2, followed by activating the CaMKKb/phosphorylated Ser431LKB1/phosphorylated Thr172AMPK/PPAR*α* pathway, thus decreasing oxidative stress and apoptosis and improving endothelial dysfunction [133]. Two other compounds derived from RA (astragaloside II and isoastragaloside I) can activate AMPK via the increase of adiponectin secretion in db/db mice [134]. Therefore, increasing the secretion of adiponectin may be an important way for RA to treat DN.

Another study on db/db mice demonstrated that AS-IV reduced glomerular mesangial matrix, proximal tubular area, and urinary NAG (a marker of proximal tubular injury) excretion by modulating the mitochondrial quality control network, as evidenced by the downregulation of the levels of mitochondrial fission-associated protein including Drp-1, Fis-1, and MFF and mitophagy-associated protein including PTEN-induced putative kinase 1 (PINK1), Parkin, p-Parkin (Ser 65), and LC-3II [135].

**Figure 2:**
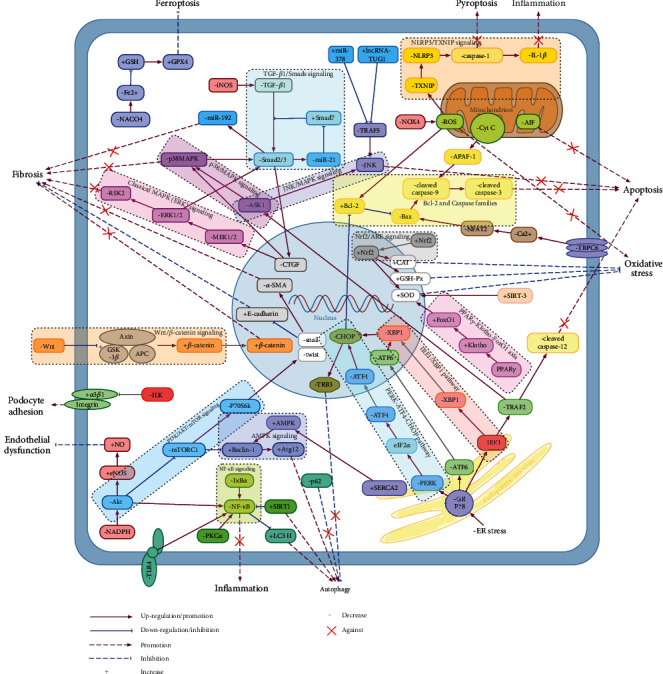
Main molecular mechanisms of RA against DN. RA and its extracts demonstrate the renal protective effect in DN through the inhibition of fibrosis, oxidative stress, endoplasmic reticulum stress, inflammation, cell apoptosis, endothelial dysfunction, and promotion of autophagy, podocyte adhesion, and so on. These actions are achieved by the regulation of Bcl-2 and caspase families and signaling pathways including TGF-*β*1/Smads, Nrf2/ARE, MAPK/ERK, p38/MAPK, JNK/MAPK, Wnt/*β*-catenin, PI3K/AKT/mTOR, AMPK, NF-*κ*B, PERK-ATF4-CHOP, IRE*α*/XBP1, and PPAR*γ*-Klotho-FoxO1.

The pharmacological effects and targets of RA and its representative extracts on DN are summarized in Table 2 and Figure 2.

## 4. Traditional Views and the Link With Pharmacological Properties of RA

In TCM theory, qi is the source of energy, constituting the human body and sustaining its life activity, exerting a positive regulatory effect on various life activities. The qi-tonifying efficacy of RA has long been recognized, since the *Shennong Materia Medica Sutra*, which was completed in the Eastern Han Dynasty (25-220 AD), has described RA as “tonifying deficiency, curing illness of children, treating exogenous diseases, and cutaneous infection.” In later generations, based on the qi-tonifying efficacy, the indication of RA extended to various syndromes involving the pathogenesis of qi-deficiency, including strengthening the body, consolidating the defensive function of the body (for treating fatigue, spontaneous sweating, loss of appetite, etc.), facilitating dispelling pathogenic qi (for treating exogenous diseases and cutaneous infection), propelling blood (for treating numbness and pain in limbs) and body fluid (for treating edema), and preventing the leakage of essences (for treating diarrhea, polyuria, proteinuria, and hematuria). Based on these traditional uses, RA has been much more in-depth applied. Its anti-inflammatory, antioxidation, anticancer, immunomodulation, and antiviral mechanisms can be related to the positive regulation of qi on the human body, thereby exerting the effects of antiaging, antidiabetes, heart protection, renal protection, liver protection, and neuroprotection [7, 136, 137].

TCM regards heat impairing qi and yin as the basic pathogenesis of DM, and the qi-deficiency is persistent in the course of DN from the beginning to the end stage. With the development of DN, qi-deficiency gradually becomes more severe, damaging other physiological functions, such as propelling body fluid, preventing the leakage of urine protein, and excreting toxins. Modern studies support that qi-deficiency syndrome is closely related to immune dysfunction, oxidative stress, inflammation, abnormalities in energy metabolism, and so on [138]. In the qi-deficiency animal models, the contents of eNOS and NO and SOD activity decreased, and the content of MDA and inflammatory factors like IL-1*β*, IFN-*γ*, IL-6, IL-8, and TNF-*α* increased [138]. These findings coincide with the changes in the progression of DN.

The components of another typical CHM with the primary efficacy of qi-tonifying, Panax ginseng C.A. Mey. (Renshen), have been proven to show similar pharmacological properties to the RA extracts, such as regulating oxidative stress, inflammation, autophagy, and apoptosis [139] and increasing NO synthesis [140]. Some TCM prescriptions or herbal pairs with qi-tonifying as the core function also suggest the potential mechanism of the qi-tonifying effect. Powder for restoring pulse beating (Shengmai Powder), decoction of four mild drugs (Sijunzi Decoction), and other TCM prescriptions for qi-tonifying, as well as commonly used herbal pairs such as ginseng and RA, have been proven to be able to regulate Akt signaling in diabetic-related cognitive decline models and reverse memory deficits in diabetic rats [141]. In the ischemic heart disease model, these prescriptions or herbal pairs can regulate AMPK, IL-6 [142], and TNF signaling [143] and upregulate Nrf2 expression, inhibiting JNK phosphorylation and cardiomyocyte apoptosis [144]. In Alzheimer's disease, they can regulate IL-1*β* and glycogen synthase kinase-3*β* (GSK-3*β*) signaling and mitigate the oxidative damage of nerve cells [145]. In chronic kidney disease (CKD), they can enhance mitochondrial energy generation and antioxidant status; eliminate free radicals; protect renal cells; improve renal microcirculation; and reduce Scr, BUN, and urinary protein [146]. Based on the above findings, the mechanisms and effects of qi-tonifying can correspond to the mechanisms and effects of RA and its extracts for treating DN.

By virtue of its superior qi-tonifying efficacy, RA positively affects DN from the perspective of both TCM and modern medicine. The effectiveness of medication based on TCM theory has been recognized by clinical and laboratory research, which confirms TCM theory. Therefore, treating DN with the qi-tonifying method deserves more clinical practice and further corroboration.

## 5. AEs and Toxicity of RA

The AEs and toxicity of RA have been studied. In most in vitro studies, the concentrations of AS-IV were under 100 *μ*mol/L, and no evidence of cytotoxicity was shown, while AS-IV 200 *μ*mol/L could decrease cell viability [77, 79]. When treated with calycosin at doses above 10 *μ*mol/L, a decrease in cell viability was observed [105]. As for in vivo experiments, feeding experiments using APS and total flavonoids showed no obvious AEs, and toxicity tests of RA total glucoside, APS, and astragalus saponins revealed that the safety dosage ranges of these components are more than 35 times the dose of humans [136].

Some clinical trials also reported some AEs of RA. In a meta-analysis of clinical studies using RA preparations to treat DN, 20 out of 66 studies reported safety outcomes [14]. Among the 376 participants in the five studies that reported AEs, there were 18 cases of dry cough (16 cases were treated with benazepril, and symptoms relieved without treatment within 2 weeks), 15 cases of Scr elevated more than 30% from baseline (Scr went down with treatment later in 13 cases), 13 cases of dizziness, three cases of angioedema, and a case of hyperkalemia. Since all the studies took ACEi/ARB as the initial therapy, which may lead to all the AEs aforementioned, and no statistical difference in the incidences of each AE was found between the treatment group and control group, the AEs of RA itself seem insignificant. However, in consideration of the data of these five studies, the facticity of the safety outcomes of the other 15 studies which reported no AEs needs to be questioned. According to the results summarized in Table 1, the AEs of RA preparations have received more attention in recent years. These outcomes indicate that serious AEs have not been found in the treatment of DN with RA preparations and, at the same time, throw light on inadequate attention to the safety evaluation of RA preparations in clinical studies. In addition, there is currently insufficient clinical evidence to evaluate the risks and benefits of the use of RA preparations in patients with ESRD. More convincing evidence is still needed.

## 6. Discussion and Prospect

The current treatment strategy for DN is still insufficient. In addition to glycemic control, a few drugs, like SGLT2 inhibitors, glucagon-like peptide 1 receptor agonists (GLP-1 RAs), ACEI/ARBs, and finerenone, have been proven to be protective of renal function [147], while side effects are not rare in the application of these drugs, for example, risk of genital infection and diabetic ketoacidosis (DKA) for SGLT2 inhibitors [148]; gastrointestinal events and risk of thyroid C-cell tumors and injection site reactions for GLP-1 RAs [149]; cough, functional renal insufficiency, hyperkalemia, angioedema, and so on for ACEI/ARBs [150]; and hyperkalemia for finerenone [4, 151]. Much less, renal insufficiency itself is the reason that restricts the use of many drugs. Despite the fact that all these approaches can only slow down but not halt the progression of DN, herbal medicines with composite compounds exhibit efficacy in a multitarget, multipathway manner compared with these chemicals and should be given full focus.

Among the aforementioned extracts, the pharmacological effects of AS-IV are achieved through the mechanisms of antioxidant, anti-inflammatory, anti-ER stress, antiapoptosis, antipyroptosis, antifibrosis, and immune regulation, improving endothelial dysfunction, promoting peripheral nerve regeneration, and limiting lipid deposition [8]. APS shows the abilities of immune regulation, anti-inflammation, antioxidant, and anti-ER stress, promoting endothelial cell proliferation, inhibiting liver glycosylation, inhibiting islet *β* cell apoptosis, and increasing insulin sensitivity in peripheral tissues [10, 152]. Calycosin exhibits effects of anti-inflammatory, antioxidant, antipyroptosis, and antiferroptosis, promoting osteogenesis, endothelial cell proliferation, and vascular dilation [11, 12]. The pharmacological mechanisms of these components, which are widely studied, partially overlap, including anti-inflammatory and antioxidation and improving vascular endothelial function. In addition, AS-IV and calycosin have been proven to be able to antipyroptosis and antifibrosis. AS-IV and APS can both exert anti-ER stress, antiapoptosis, antibacterial, antiviral, and immune-regulation effects. The differences among the pharmacological effects of each component may provide new perspectives for drug development for different diseases or pathological processes. Among its representative components, AS-IV, as the constituent for quality evaluation in the *Chinese Pharmacopeia*, gains the most interest from researchers in the DN field, while APS, calycosin-7-O-*β*-D-glucoside, calycosin, and other components are relatively less investigated. Based on its comprehensive effects of antioxidation, anti-inflammatory, modulating regulated cell death, anti-ER stress, antifibrosis, and improving endothelial dysfunction, AS-IV could be priorly considered to be developed in the treatment of DN. However, the therapeutic effects for other diseases of APS calycosin-7-O-*β*-D-glucoside and calycosin also suggest their potential pharmacological mechanisms on DN, and more evidence remains to be explored to provide new strategies for the treatment of DN in the future.

Except for these representative components, network pharmacology studies have found many other flavonoids in RA with multitudinous targets against DN, including quercetin, formononetin, kaempferol, 7-Omethylisomucronulatol, and isorhamnetin [153]. Kaempferol acts its renal protective effects through the anti-inflammation, antioxidation, and antifibrosis mechanisms [154–157]. The pharmacological effects of quercetin are similar to kaempferol, which acts on endothelium, TECs, MCs, and podocytes to alleviate DN [158–162]. These protective effects are reflected in improvements in the renal index, urine protein, uric acid, urine ALB, and Scr levels [163]. Formononetin can also alleviate oxidative stress, restore mitochondrion function, and inhibit renal fibrosis in DN models [164–167]. Isorhamnetin, as an anti-inflammatory and antioxidation agent, shows a definite antidiabetic effect and the ability to modulate autophagy in renal tissues [168, 169]. These components share common pharmacological mechanisms with the representative components of RA. Together, the multiple components of RA act in coordination with each other and contribute to the treatment of DN.

## 7. Conclusion

Based on the present findings, RA and its extracts have a multiscale act on various mechanisms of DN as well as in histopathological changes in most parts of the nephron. Besides, these components show definite protection effects on renal function. Specific mechanisms include inhibition of oxidative stress, inhibition of ER stress, modulation of regulated cell death, anti-inflammation, and inhibition of renal fibrosis. Although many studies have elucidated the therapeutic effect of RA on DN through histopathological changes, clinically, biopsy is not the prior choice of DN patients. The clinical efficacy evaluation still relies on the detection of biochemical indexes. The histopathological examination reflects structure, while biochemical index detection focuses on function, which is what truly matters for patients. The scarcity and low quality of clinical trials failed to provide sufficient evidence to prove how much RA can improve renal function and how safe the different types of RA preparations are, which hiders the usage of RA in clinical practice. It is worth noting that researchers have developed mediums that can encapsulate active extracts of herbal medicine including RA, enhancing the solubility and sustained release, making it possible to promote stable and highly bioavailability herbal ingredient preparations to clinical practice [83, 170, 171]. Further, strictly designed large-scale, long-term follow-up trials are warranted to provide definitive evidence for the clinical efficacy of RA and its extracts. Still, these findings pave the way for the future development of RA and its extracts as potential therapeutic preparations in the management of DN.

## Figures and Tables

**Table 1:** Study characteristics of the recent clinical researches of RA in treating DN.

**Ref.**	**Baseline renal function or DN stage**	**Participants (T/C)**	**Average age (T/C)**	**Average history of DM (T/C)**	**RA preparations, dose, and frequency**	**Basic therapeutics, dose, and frequency**	**Treatment duration**	**Treatment effects of RA**	**Incidence of adverse effects (T/C)**	**Adverse effects of treatment group**
Chen et al. [16]	Mogensen IV	32/32	54.18/53.76	9.06/9.16	Tablet, 2.2 g, bid	Irbesartan 150 mg, qd; hydroxychloroquine 200 mg, bid	12 weeks	Scr, BUN, eGFR, 24 h UP ↓	18.75%/12.5%	4 cases of gastrointestinal reactions and 2 cases of liver function damage
Zhang et al. [17]	Mogensen III	50/50	40.7/40.9	7.5/7.3	APS injection 250 mg, qd	Conventional therapy	2 weeks	Scr, BUN ↓	NS	NS
Wang, Liu, and Guo [18]	Microalbuminuria, UACR ≤300 mg/g	32/30	59.5/58.1	10.26/9.25	Granules, 4.5 g, qd	Conventional therapy	3 weeks	UACR ↓	NS	NS
Liu [19]	NS	25/25	74.63/73.86	12.11/12.21	Decoction with RA 30 g, qd	Conventional therapy	1 month	mALB, UAER, Scr ↓	NS	NS
Zhou et al. [20]	Mogensen III–IV	40/40	58.12/57.88	6.17/6.88	APS injection 250 mg, qd	Conventional therapy	1 week	UAER, 24 h UP, Scr, BUN ↓	No AEs	No AEs
Sheng and Chen [21]	Mogensen III	40/40	48.14/46.78	7.63/8.16	Tablet, 2.2 g, bid	Irbesartan 150 mg, qd	12 weeks	UAER ↓	12.5%/7.5%	2 cases of hyperkalemia, 2 cases of gastrointestinal reactions, and 1 case of rash
Han [22]	NS	35/35	53.13/53.51	12.23/12.02	Injection, 20 mL, qd	Conventional therapy	4 weeks	Scr, BUN ↓	2.9%/2.9%	1 case of nausea
Xu [23]	UAER>300 mg/24 h	50/50	57.8/58.1	9.4/9.2	Injection, 30 mL, qd	Benazepril 10 mg, qd	8 weeks	Scr, BUN, 24 h UP, mALB ↓	10%/14%	2 cases of dizziness, 2 cases of cough, and 1 case of headache
Sun [24]	NS	42/42	57.40/57.62	9.28/8.76	Injection, 40 mL, qd	Calcium dobesilate 0.5 g, tid	2 weeks	Scr, BUN, UAER ↓	NS	NS
Chen et al. [25]	Mogensen III	42/43	61.3/61.8	NS	Granules, 4 g, bid	Losartan 100 mg, qd	12 weeks	24 h UP, UACR ↓	NS	NS
Xu and Liu [26]	Mogensen III	43/43	58.4/59.2	9.8/10.2	Injection, 30 mL, qd	Alprostadil injection 20 *μ*g, qd	4 weeks	Scr, BUN, UAER, urine *β*2-MG ↓	2.3%/18.6%	1 case of allergy
Wang and Du [27]	NS	26/26	39.4/56.5	3.5/3.6	Granules, 15 g, bid	Valsartan 80 mg, qd	3 weeks	Scr, 24 h UP, mALB ↓	NS	NS
Wang and Zhang [28]	Mogensen III	35/35	54.47/51.65	7.06/6.78	Injection, 50 mL, qd	Atorvastatin 20 mg, qd	3 months	SCr, UAER, TGF-*β*1 ↓, eGFR ↑	No AEs	No AEs
Wang [29]	Mogensen III	48/48	51.8/52.4	6.2/6.5	Injection, 40 mL, qd	Spironolactone 20 mg, bid	1 month	mALB, BUN, Scr, UAER, *β*2-MG ↓	4.2%/6.3%	1 case of dizziness and 1 case of cough
Sun [30]	Mogensen III	50/50	59.61/59.48	5.58/5.79	Injection, 20 mL, qd	Valsartan 80 mg, qd	2 weeks	Scr, BUN, 24 h UP ↓	No AEs	No AEs
Qiao [31]	NS	40/40	58.5/58.5	7.5/6.5	Injection, 20 mL, qd	Benazepril 10 mg, qd	4 weeks	Scr, 24 h UP, mALB ↓	NS	NS
Liu and Chen [32]	Mogensen III	60/60	55.8/55.3	12.3/12.2	Injection, 30 mL, qd	Conventional therapy	3 weeks	Cys-C, mALB, UACR, *β*2-MG, NAG ↓	No AEs	No AEs
Zhou and Zhang [33]	Mogensen III–IV	50/50	50.3/50.2	10.2/11.1	Injection, 40 mL, qd	Conventional therapy	10~15 days	24 h UP, Scr ↓, serum ALB ↑	NS	NS
Zhang [34]	Mogensen III	89/89	54.8/55.1	4.9/5.1	Injection, 20 mL, qd	Valsartan 80 mg, qd	3 weeks	Scr, BUN, urine *β*2-MG, mALB ↓, serum ALB ↑	NS	NS
Wang [35]	NS	20/20	64.13/64.25	1.39/1.24	Granules, 4 g, bid	Valsartan 80 mg, qd	3 months	24 h UP, Scr ↓	NS	NS
Qi [36]	NS	70/68	39/40	NS	Injection, 30 mL, qd	Captopril 12.5 mg, bid	4 weeks	24 h UP ↓, serum ALB ↑	NS	NS
Li and Xu [37]	Mogensen III	55/55	NS(48~73)	7.8/7.9	Injection, 50 mL, qd	Irbesartan 150 mg, qd	1 month	Scr, BUN, UAER, 24 h UP ↓	NS	NS
Li [38]	NS	43/43	54.7/54.0	7.3/7.5	Injection, 20 mL, qd	Benazepril 5 mg, qd	4 weeks	Scr, BUN, UAER ↓	4.7%/7.0%	1 case of dizziness and 1 case of cough
He et al. [39]	UAER>30 mg/24 h, Scr ≤350 *μ*mol/L	18/18	51/50	NS	Injection, 20 mL, qd	Captopril 12.5~100 mg/day, frequency not specific	12 weeks	24 h UP ↓	No AEs	No AEs
Y. Zhang and H. Zhang [40]	NS	30/30	47.6/46.9	NS	Injection, 40 mL, qd	ACEi, details not specific	4 weeks	UAER ↓	No AEs	No AEs
Xiang [41]	Mogensen III	34/33	57.52/58.42	NS	Granules, 4 g, bid	Valsartan 80 mg, qd	3 months	mALB, 24 h UP, UAER, Cys-C, *β*2-MG, NAG ↓	No AEs	No AEs
Tang [42]	Mogensen III	72/72	52.48/52.13	NS	Injection, 20 mL, qd	Valsartan 80 mg, qd	4 weeks	Scr, BUN, 24 h UP, *β*2-MG ↓	NS	NS
Ren [43]	Mogensen III	45/45	57.6/56.9	6.7/6.6	Injection, 50 mL, qd	Irbesartan 150 mg, qd	1 month	Scr, Ccr, 24 h UP, UAER, *β*2-MG ↓	NS	NS
Liu and Deng [44]	NS	37/37	60.2/56.7	9.8/10.6	Injection, 20 mL, qd	Valsartan 80 mg, qd	3 weeks	Scr, 24 h UP, mALB ↓	NS	NS
Li [45]	UAER>30 mg/24 h	23/23	56.9/56.2	6.7/6.2	Injection, 40 mL, qd	Enalapril 10 mg, qd	2 weeks	Scr, BUN, 24 h UP, UAER ↓	13%/17.4%	2 cases of cough and 1 case of headache
Fang et al. [46]	24 h UP>3.5 g, serum ALB<30 g/L, Scr<354 *μ*mol/L	40/40	61.9	12.1	Injection, 30 mL, qd	Alprostadil injection 10 *μ*g, qd	2 weeks	Scr ↓, serum ALB ↑, UAER ↓	NS	NS
Cai and Cui [47]	Mogensen III	40/40	56.5/57.2	7.8/7.9	Injection, 50 mL, qd	Benazepril 10 mg, qd	4 weeks	mALB, 24 h UP, UAER, *β*2-MG ↓	NS	NS
Xiao and Liu [48]	Mogensen III	45/45	47.5/48.3	10.6/11.7	injection, 30 mL, qd	Alprostadil injection 20 *μ*g, qd	4 weeks	Scr, BUN, UAER, urine *β*2-MG ↓	2.2%/0	1 case of allergy
Wang [49]	NS	54/54	68.1/68.5	7.4/7.2	Injection, 50 mL, qd	Irbesartan 150 mg, qd	1 month	Scr, BUN, 24 h UP, UAER ↑	NS	NS
Shi and Lin [50]	24 h UP 0.3~1 g	42/42	42.98	1.85	Injection, 40 mL, qd	Valsartan 80 mg, qd	1 week	Scr, BUN, mALB ↓	NS	NS
Ouyang [51]	Mogensen IV	64/64	53.4/52.8	6.8/7.0	Injection, 30 mL, qd	Atorvastatin 20 mg, qd	4 weeks	Scr, 24 h UP, CRP ↓	NS	NS
Luo [52]	NS	50/50	58.6/59.1	11.3/11.5	Injection, 20 mL, qd	Valsartan 80 mg, qd or bid	2 weeks	Scr, BUN, *β*2-MG ↓	No AEs	No AEs
Liu, Meng, and Wei [53]	Mogensen III	36/36	54.0/53.8	6.4/6.3	Injection, 50 mL, qd	Benazepril 10 mg, qd	1 month	UAER ↓	No AEs	No AEs
Liu et al. [54]	Mogensen III	28/28	67.0/67.5	7.3/7.2	Injection, 40 mL, qd	Irbesartan 150 mg, qd	4 weeks	Scr, UAER ↓	NS	NS
Li and Zhang [55]	Mogensen III-IV	30/30	41.2/40.8	NS	Injection, 40 mL, qd	Benazepril 10 mg, qd	4 weeks	UAER ↓	NS	NS
Kong [56]	NS	39/39	52.8/53.2	8.2/8.6	Injection, 20 mL, qd	Benazepril 10 mg, qd	3 weeks	Scr, BUN ↓	NS	NS
Chen [57]	UAER>30 mg/24 h	50/50	63.15/63.23	6.32/6.25	Injection, 30 mL, qd	Telmisartan (dose not given)	2 weeks	Scr, BUN ↓	NS	NS
Chen [58]	Mogensen III	34/34	48.89/49.06	7.65/8.26	Injection, 20 mL, qd	Conventional therapy	4 weeks	UAER ↓	NS	NS
Zhao [59]	NS	35/35	52.7/51.4	8.2/7.9	Injection, 1.5~1.8 mL/(kg·d)	Conventional therapy	2 months	Scr, BUN ↓	NS	NS
Zhang [60]	Mogensen III	28/28	51.25/51.41	9.42/9.41	Injection, 60 mL, qd	Losartan 50 mg, qd	4 weeks	Scr, BUN, UAER ↓	NS	NS
Zhang and Cao [61]	NS	30/30	55/59	9.0/8.5	Injection, 40 mL, qd	Benazepril 10 mg, qd	3 weeks	Scr, 24 h UP, mALB ↓	NS	NS
Sun, Zhao, and Ding [62]	Mogensen III	38/38	52.63/51.84	6.1/5.9	Injection, 20 mL, qd	Telmisartan 40 mg, qd	2 weeks	UAER ↓	NS	NS
Dou and Wang [63]	Mogensen III	24/24	51/54	12/11	Tablet, 2.2 g, bid	Valsartan 80 mg, qd	12 weeks	24 h UP ↓	NS	NS
Deng et al. [64]	Mogensen III	36/30	80.02/78.02	NS	APS injection 250 mg, qd	Conventional therapy	3 weeks	UAER, 24 h UP, Cys-C ↓	NS	NS
Chang et al. [65]	24 h UP>2 g	24/24	54.14/56.38	11.72/11.92	Granules, 4 g, bid	Tripterygium glycosides 20 mg, tid	12 weeks	24 h UP, serum ALB, CRP ↓	4.2%/25%	1 case of liver function damage

*Note:* Symbols “↑” and “↓” represent increase and decrease, respectively.

Abbreviations: 24 h UP, 24-h urinary protein; *β*2-MG, *β*2-microglobulin; ALB, albumin; BUN, blood urea nitrogen; CRP, C-reactive protein; Cys-C, serum cystatin C; NAG, N-acetyl-*β*-D aminoglucosidase; mALB, microalbumin; Scr, serum creatinine; UACR, urinary albumin to creatinine ratio; UAER, urinary albumin excretion rate.

**Table 2:** Prevention mechanism of RA on diabetic nephropathy.

	**Model class**	**Modeling method**	**Pharmacological effect**	**Targets**	**Ref.**
AS-IV	db/db mice	/	Ameliorate glomerular hypertrophy and mesangial hyperplasia, protect the podocytes, attenuate proteinuria, and protect renal function	Activate PPAR*γ*-Klotho-FoxO1 axis and inhibit oxidative stress	Xing et al. [69]
	Mouse podocytes	HG	Protect the podocytes		

AS-I	SD rats	STZ	Reduce ECM deposition, alleviate GBM thickening, ameliorate mesangial hyperplasia, attenuate proteinuria, and reduce blood glucose	Downregulate hyperglycemia-oxidative stress-AGEs/TGF-*β*1 pathway and inhibit oxidative stress	Yin et al. [70]

AS-IV	Male SD rats	STZ	Reduce ECM deposition, ameliorate mesangial hyperplasia, alleviate GBM thickening, protect the podocytes, and attenuate proteinuria	Inhibit MAPK/ERK pathway, upregulate TRPC6 expression, and inhibit oxidative stress	He et al. [71]

AS-IV	C57BL/6J mice	STZ	Reverse the increase of glomerular surface area and expansion of mesangial matrix, alleviate podocyte apoptosis, upregulate nephrin and podocin expression, and alleviate renal injury and mitochondrial disorder	Ameliorate mitochondrial dysfunction by upregulating Nrf2-ARE/TFAM signaling	Shen et al. [72]
	Mouse podocytes	HG	Inhibit podocyte mitochondrial morphological alterations and podocyte apoptosis		

AS-IV	Male SD rats	STZ	Ameliorate mesangial hyperplasia, protect the podocytes, and attenuate proteinuria	Modulate caspase and Bcl-2 protein families and inhibit oxidative stress	Gui et al. [73]
	Mouse podocytes	HG	Protect the podocytes		

Calycosin	NRK-52E cells	HG	Recover cell viability and membrane damage	Regulate Sirt3/SOD2/caspase-3 signaling and inhibit oxidative stress	Jiang et al. [81]

Calycosin	Male SD rats	STZ	Recover mitochondrial viability	Mitigated lipoperoxidation of the cell membrane and oxidative stress-triggered mitochondrial respiration dysfunction	Huang et al. [83]

AS-I	Rat mesangial cells	HG	Reduce ECM deposition and decrease HbA1c levels	Increase activities of antioxidant enzymes, inhibit iNOS/TGF-*β*1 signaling, and inhibit oxidative stress	Yin et al. [74]

AS-IV	Human mesangial cell	HG	Ameliorate mesangial hyperplasia and improve MC contractile function	Inhibit the NADPH oxidase/ROS/Akt/NF-*κ*B pathway and inhibit oxidative stress	Sun et al. [75]

AS-IV	NRK-52E cell	GA	Inhibited EMT	Increase activities of antioxidant enzymes and inhibit oxidative stress	Qi et al. [76]

AS-IV	HK-2 cells	HG	Protect TECs from apoptosis	Regulate the Nrf2/ARE signaling pathway, modulate caspase and Bcl-2 protein families, and inhibit oxidative stress	Wang and Guo [77]

AS-IV	Male SD rats	STZ	Inhibited EMT	Increase activities of antioxidant enzymes, inhibit Wnt/*β*-catenin pathway, and inhibit oxidative stress	Wang et al. [78]
	HK-2 cells	HG	Reduce ECM deposition		

AS-IV	HK-2 cells	PA	Reduce lipid deposition and protect TECs from apoptosis	Modulate caspase and Bcl-2 protein families and inhibit oxidative stress	Chen et al. [79]

Calycosin and calycosin-7-O-*β*-D-glucoside	Rat mesangial cells	HG	Ameliorate mesangial hyperplasia	/	Tang et al. [80]
	Human glomerular endothelial cells	AGEs	Protect glomerular endothelial cells from apoptosis		

Calycosin	SD rats	High-fat diet/STZ	Ameliorate glomerular hypertrophy, reduce ECM deposition, and attenuate proteinuria	Inhibit IL-33/ST2 and Nrf2/ARE signaling and inhibit oxidative stress	Elsherbiny et al. [82]

AS-IV	Male SD rats	STZ	Protect TECs from apoptosis, alleviate epithelial cell edema, alleviate GBM thickening, reduce ECM deposition, attenuate proteinuria, and protect renal function	Modulate caspase and Bcl-2 protein families and inhibit ER stress	Ju et al. [87]

AS-IV	Male SD rats	STZ	Ameliorate mesangial hyperplasia, protect the podocytes, and attenuate proteinuria	Modulate Bcl-2 protein family, downregulate PERK-ATF4-CHOP pathway, and inhibit ER stress	Chen et al. [88]

AS-IV	Male SD rats	STZ	Ameliorate mesangial hyperplasia and reduce ECM deposition	Modulate caspase protein family and inhibit ER stress	Wang et al. [89]
	Human podocytes	Tunicamycin	Protect the podocytes		

Calycosin	Male SD rats	STZ	Attenuate proteinuria, protect renal function, and alleviate interstitial collagen deposition and thickening of glomerular basement membranes	Modulated NF-*κ*B/p65/NLRP3/TXNIP-mediated pyroptotic signaling	Yosri et al. [84]

AS-IV	db/db mice	/	Attenuate proteinuria and improve renal structural changes such as mesangial proliferation, ECM deposition, podocyte foot process fusion, foot process widening, podocyte loss, and GBM thickening, accompanied by the upregulation of the podocyte markers podocin and synaptopodin	Inhibit NLRP3 inflammasome-mediated inflammation	Feng et al. [95]
	Mouse podocytes	HG	Inhibit cell pyroptotic and increase cell viability		

AS-IV	Male SD rats	STZ	Improve renal function, protect podocytes, and inhibit glomeruli pyroptosis	Increase klotho levels in serum and kidney tissue	He et al. [96]
	Mouse podocytes	HG	Inhibited pyroptosis of podocytes	Increase klotho expression via the inhibition of the NF-*κ*B/NLRP3 axis	

Calycosin	db/db mice	/	Inhibit lipid ROS production, protect renal function, and alleviate tubular dilatation, loss of proximal tubule brush border, and collagen deposition	Upregulate GSH and GPX4 levels and downregulate NCOA4 expression	Huang et al. [98]
	HK-2 cells	HG	Inhibit lipid ROS production and ferroptosis-induced tubular injury and increase cell viability		

AS-IV	C57BL/6J mice	STZ	Protect the podocytes, ameliorate glomerular hypertrophy and mesangial hyperplasia, alleviate glomerulosclerosis, attenuate proteinuria, and protect renal function	Regulate AMPK and PI3K/AKT/mTOR signaling, inhibit ER stress, and enhance autophagy	Guo et al. [90]
	Mouse podocytes	HG	Protect the podocytes		

AS-IV	db/db mice	/	Ameliorate glomerular hypertrophy and mesangial hyperplasia, protect the podocytes, attenuate proteinuria, protect renal function, normalize glucose tolerance and insulin sensitivity, and alleviate hypertension	Modulate caspase and Bcl-2 protein families and inhibit ER stress	Guo et al. [91]
	Mouse podocytes	PA	Protect the podocytes		

AS-IV	Male KKAy mice	High-fat diet	Ameliorate mesangial hyperplasia and reduce ECM deposition	Inhibit NF-*κ*B pathway, anti-inflammation, and upregulate autophagy-related proteins	Wang et al. [100]
	Mouse glomerular mesangial cells	HG	Reduce ECM deposition and ameliorate mesangial hyperplasia		

AS-IV	Male SD rats	STZ	Reduce ECM deposition, ameliorate mesangial hyperplasia, protect the podocytes, and attenuate proteinuria	Inhibit NF-*κ*B pathway, anti-inflammation	Gui et al. [102]

APS	SD rats	STZ	Protect renal function and ameliorate mesangial cell hyperplasia and glomerular basement membrane thickening	Inhibit TLR4/NF-*κ*B pathway, anti-inflammation	Guo et al. [103]
	MPC5 cells	HG	Alleviate podocyte proliferation		

APS	SD rats	STZ	/	Inhibit NF-*κ*B pathway, anti-inflammation	Zhang, Wu, and Cheng [104]

Calycosin	db/db mice	/	Reduce ECM deposition and alleviate GBM thickening and glomerular sclerosis	Inhibit NF-*κ*B pathway, anti-inflammation	Zhang et al. [105]
	Mouse tubular epithelial cells	AGEs	/		

Astragalosides	MCs	HG	Reduce ECM deposition and ameliorate mesangial hyperplasia	Regulate TGF-*β*1/Smad signaling	Chen et al. [113]

AS-IV	SD rats	STZ	Reduce ECM deposition and alleviate GBM thickening and glomerular atrophy	Modulate the TGF-*β*1/Smad/miR-192 signaling pathway, antifibrosis	Mao et al. [114]
	Rat MCs	HG	Ameliorate mesangial hyperplasia		

AS-IV	Male KKAy mice	High-fat diet	Protect the podocytes, alleviate GBM thickening, reduce ECM deposition, antifibrosis, and attenuate proteinuria	Regulate TGF-*β*1/Smad signaling, antifibrosis	Wang et al. [115]
	Mouse MCs	HG			

AS-IV	Human tubular endothelial cells	HG	Protect TECs from apoptosis	Regulate TGF-*β*1/Smads and p38/MAPK signaling, antifibrosis	Wang et al. [116]

AS-IV	HK-2 cells	HG	Reduce ECM deposition and inhibit tubular EMT	Inhibit mTORC1/p70S6K signaling, antifibrosis	Chen et al. [117]

AS-IV	Mouse renal fibroblasts	TGF-*β*1	Reduce ECM deposition and decrease fibroblast viability	Inhibit the MAPK and NF-*κ*B signaling pathways, antifibrosis	Che et al. [118]

Astragaloside	Male KKAy mice	High-fat diet	Ameliorate mesangial hyperplasia, alleviate TEC cytoplasm vacuole degeneration, and reduce renal interstitial inflammatory cells	Regulate TGF-*β*1/Smad signaling, antifibrosis	Wang et al. [119]

AS-IV	db/db mice	/	Attenuate proteinuria, protect renal function, and improve proximal renal tubular cell swollen, renal tubular epithelial cells vacuolated, collagen deposition, glomerular hypertrophy, and mesangial matrix expansion	Decrease the expression of CX3CL1 and inhibit the activation of the RAF/MEK/ERK pathway	Hu et al. [112]
	HK-2 cells	HG	Suppress vimentin and *α*-SMA expression, increase E-cadherin expression, and suppress EMT		

AS-IV	Male C57BL/6 mice	STZ	Ameliorate mesangial hyperplasia, protect the podocytes, attenuate proteinuria, and protect renal function	Inhibit MEK1/2-ERK1/2-RSK2 signaling, antifibrosis	Song et al. [120]

AS-IV	db/db mice	/	Alleviate GBM and TGM thickening, protect the podocytes, reduce ECM deposition, and attenuate proteinuria	Inhibit AKT/mTOR, NF-*κ*B, and MAPK/ERK pathways, antifibrosis	Sun et al. [121]

AS-IV	Male SD rats	STZ	Reduce ECM deposition, attenuate proteinuria, protect renal function, and decrease HbA1c levels	Activate eNOS and NO	Fan et al. [123]
	HRGECs	HG	Protect glomerular endothelial cells from apoptosis		

AS-IV	Mouse podocyte cells	HG	Protect the podocytes	Upregulate *α*3*β*1 integrin and inhibit ILK	Chen et al. [124]

AS-IV	Male SD rats	STZ	Protect the podocytes and attenuate proteinuria	Upregulate *α*3*β*1 integrin and inhibit ILK	Chen et al. [125]

AS-IV	Mouse podocytes	HG	Protect the podocytes	Downregulate TRPC6 and modulate Bcl-2 protein family	Yao et al. [126]

AS-IV	Sprague–Dawley rats	STZ	Protect the podocytes, attenuate proteinuria, protect renal function, and reduce blood glucose and arterial blood pressure	Regulate miR-378/TRAF5 signaling pathway	Lei et al. [128]
	MPC5 cells	HG	Protect the podocytes		

AS-IV	Sprague–Dawley rats	STZ	Attenuate proteinuria	Regulate lncRNA-TUG1/TRAF5 signaling pathway and modulate caspase protein family	Lei et al. [129]
	MPC5 cells	HG	Protect the podocytes		

AS-IV	db/db mice	/	Ameliorate mesangial hyperplasia and attenuate proteinuria	Inhibit PINK1/Parkin-mediated mitophagy and modulate the mitochondrial quality control network	Liu et al. [135]

Abbreviations: *α*-SMA, *α*-smooth muscle actin; AGEs, advanced glycation end products; Akt, protein kinase B; AMPK, adenosine 5′-monophosphate (AMP)-activated protein kinase; APSs, astragalus polysaccharides; AS-IV, astragaloside IV; ATF4/6, activating transcription factor 4/6; Bcl-2, B-cell lymphoma-2; Caspase, cysteinyl aspartate specific proteinase; CHOP, C/EBP-homologous protein; CX3CL1, chemokine C-X3-C-motif ligand 1; ECM, extracellular matrix; EMT, epithelial-mesenchymal transition; eNOS, endothelial nitric oxide synthase; ERK, extracellular signal-regulated kinase; ER stress, endoplasmic reticulum stress; FoxO1, forkhead box transcription factor O1; GA, glycosylated albumin; GBM, glomerular basement membrane; GPX4, phospholipid peroxidase glutathione peroxidase 4; GSH, glutathione; HbA1c, glycosylated hemoglobin; HFD, high-fat diet; HRGECs, human renal glomerular endothelial cells; iNOS, inducible nitric oxide synthase; LK, integrin-linked kinase; lncRNA-TUG1, long noncoding RNA taurine upregulated 1; MAPK, mitogen-activated protein kinase; MCs, mesangial cells; MEK1/2, mitogen-activated protein kinase kinase 1/2; miR-21/192/378, microRNA 21/192/378; MPC5 cells, murine podocyte cell line; mTOR, mammalian target of rapamycin; NADPH, nicotinamide adenine dinucleotide phosphate; NCOA4, nuclear receptor coactivator 4; NF-*κ*B, nuclear factor-*κ*B; NLRP3, NOD-like receptor thermal protein domain associated protein 3; NO, nitric oxide; Nrf2, NF-E2-related factor 2; PA, palmitic acid; PERK, protein kinase R (PKR)-like endoplasmic reticulum kinase; PINK1, PTEN-induced putative kinase 1; PI3K, phosphatidylinositol 3-kinase; PPAR*γ*, peroxisome proliferators-activated receptor *γ*; RAF, Raf protein kinase; ROS, reactive oxygen species; RSK2, ribosomal protein S6 kinase 90 kDa polypeptide 3; SIRT1/3, sirtuin1/3; Smad, drosophila mothers against decapentaplegic protein; SOD, superoxide dismutase; TECs, tubular epithelial cells; TFAM, mitochondrial transcription factor A; TGF-*β*, transforming growth factor-*β*; TLR4, Toll-like receptor 4; TRAF2/5, TNF receptor–associated factor 2/5; TRPC6, transient receptor potential channel-6; TXNIP, thioredoxin-interacting protein.

## Data Availability

The data used to support the findings of this study are available from the corresponding author upon request.

## Nomenclature

24 h UP: 24-h urinary protein
*α*-SMA: *α*-smooth muscle actin
*β*2-MG: *β*2-microglobulin
AEs: adverse effects
AGEs: advanced glycation end products
AIF: apoptosis-inducing factor
Akt: protein kinase B
ALB: albumin
AMPK: adenosine 5′-monophosphate (AMP)-activated protein kinase
Ang II: angiotensin II
APAF-1: apoptotic protease activating factor-1
APC: adenomatous polyposis coli
APSs: astragalus polysaccharides
ASC: apoptosis-associated speck-like protein
AS-IV: astragaloside IV
ASK1: apoptosis signal-regulating kinase 1
ATF4/6: activating transcription factor 4/6
Atg12: autophagy-related protein 12
Axin: axis inhibition protein
Bax: Bcl2-associated X protein
Bcl-2: B-cell lymphoma-2
BUN: blood urea nitrogen
CaMKKb: calcium/calmodulin-dependent protein kinase kinase
Caspase-3/9/12: cysteinyl aspartate specific proteinase-3/9/12
CAT: catalase
Ccr: creatinine clearance rate
CHM: Chinese herbal medicine
CHOP: C/EBP-homologous protein
CKD: chronic kidney disease
Col-1: Type 1 collagen
Col-IV: Type IV collagen
CRP: C-reactive protein
CTGF: connective tissue growth factor
CX3CL1: chemokine C-X3-C-motif ligand 1
Cys-C: serum cystatin C
Cyt C: cytochrome C
DKA: diabetic ketoacidosis
DKD: diabetic kidney disease
DM: diabetes mellitus
DN: diabetic nephropathy
ECM: extracellular matrix
eGFR: estimated glomerular filtration rate
eIF2*α*: eukaryotic initiation factor 2*α*
EMT: epithelial-mesenchymal transition
eNOS: endothelial nitric oxide synthase
ER stress: endoplasmic reticulum stress
ERK1/2: extracellular signal-regulated kinase 1/2
ESRD: end-stage kidney disease
FBG: fasting blood glucose
FN: fibronectin
FoxO1: forkhead box transcription factor O1
GA: glycosylated albumin
GBM: glomerular basement membrane
GECs: glomerular endothelial cells
GLP-1 RAs: glucagon-like peptide 1 receptor agonists
GPX4: phospholipid peroxidase glutathione peroxidase 4
GRP78: glucose-regulated protein 78 kD
GSH: glutathione
GSH-Px: glutathione peroxidase
GSK-3*β*: glycogen synthase kinase-3*β*
HbA1c: glycosylated hemoglobin
HFD: high-fat diet
HGF: hepatocyte growth factor
IFN-*γ*: interferon-*γ*
ILK: integrin-linked kinase
iNOS: inducible nitric oxide synthase
IRE1: inositol requiring enzyme 1
I*κ*B*α*: inhibitor of NF-*κ*B
JNK: c-Jun N-terminal kinase
LC3: microtubule-associated protein 1 light chain 3
lncRNA-TUG1: long noncoding RNA taurine upregulated 1
mALB: microalbumin
MAPK: mitogen-activated protein kinase
MCP-1: monocyte chemoattractant protein-1
MCs: mesangial cells
MDA: malondialdehyde
MEK1/2: mitogen-activated protein kinase kinase 1/2
miR-21/192/378: microRNA 21/192/378
MPC5 cells: murine podocyte cell line
mTORC1: mammalian target of rapamycin complex 1
NADPH: nicotinamide adenine dinucleotide phosphate
NAG: N-acetyl-*β*-D aminoglucosidase
NCOA4: nuclear receptor coactivator 4
NFAT2: nuclear factor of activated T cells 2
NF-*κ*B: nuclear factor-*κ*B
NLRP3: NOD-like receptor thermal protein domain associated protein 3
NO: nitric oxide
NOX4: NADPH oxidase 4
Nrf2: NF-E2-related factor 2
p62: autophagy-related protein p62
P70S6k: P70 ribosomal protein S6 kinase
PA: palmitic acid
PERK: protein kinase R (PKR)-like endoplasmic reticulum kinase
PI3K: phosphatidylinositol 3-kinase
PINK1: PTEN-induced putative kinase 1
PKC*α*: protein kinase C*α*
PPAR*α*/*γ*: peroxisome proliferators-activated receptor *α*/*γ*
RAF: Raf protein kinase
RCD: regulated cell death
ROS: reactive oxygen species
RSK2: ribosomal protein S6 kinase 90 kDa polypeptide 3
Scr: serum creatinine
SERCA2: sarco/endoplasmic reticulum Ca^2+^-ATPase 2
SIRT1/3: sirtuin1/3
Smad2/3/7: drosophila mothers against decapentaplegic protein2/3/7
SOD: superoxide dismutase
T-AOC: total antioxidative capability
TCM: traditional Chinese medicine
TECs: tubular epithelial cells
TFAM: mitochondrial transcription factor A
TGF-*β*: transforming growth factor-*β*
TLR4: Toll-like receptor 4
TNF-*α*: tumor necrosis factor-*α*
TRAF2/5: TNF receptor-associated factor 2/5
TRB3: tribbles homologous protein 3
TRPC6: transient receptor potential channel-6
TXB_2_: thromboxane B_2_
TXNIP: thioredoxin-interacting protein
UACR: urinary albumin to creatinine ratio
UAER: urinary albumin excretion rate
UPR: unfolded protein response
XBP1: X-box binding protein 1
